# Development and validation of multimorbidity index predicting mortality among Chinese older adults

**DOI:** 10.1093/geroni/igab046.2324

**Published:** 2021-12-17

**Authors:** Yan Luo, Zi-Ting Huang, Hui-wen Xu, zi-shuo Chen, He-Xuan Su, Hui Liu, Beibei Xu

**Affiliations:** 1 Peking University School of Public Health, Beijing, Beijing, China (People's Republic); 2 Peking university School of Public Health, Beijing, Beijing, China (People's Republic); 3 Peking University Medical Informatics Center, Beijing, Beijing, China (People's Republic)

## Abstract

This study aimed to construct a multimorbidity index among Chinese older adults. Participants aged 65-84 years (n=11,757) from the Chinese Longitudinal Healthy Longevity Survey (CLHLS). Fourteen self-reported chronic conditions were assessed at baseline. Outcome was all-cause mortality within five-year follow-up. We used restrictive association rules mining to identify the patterns of multiple chronic conditions associated with mortality. The weights of conditions and disease combinations were assigned using logistic regression adjusted by age and sex in training set. Multimorbidity index (MI) with individual diseases and multimorbidity index incorporating disease combinations (MIDC) were developed. We compared the performance of MI and MIDC with condition count and XGBoost algorithm in the validation set. There were no significant differences of c-statistics between condition count (0.687) and MI (0.692) or MIDC (0.689). The c-statistic of XGBoost algorithm (0.675) was the lowest among all models. The Integrated Discrimination Improvement (IDI) and categorical Net Reclassification Index (NRI) for MI (IDI: 0.01, P < 0.001; NRI: 0.01, P = 0.127), MIDC (IDI: 0.004, p = 0.002; NRI: 0.02, P = 0.033), and XGBoost model (IDI: 0.02, P < 0.001; NRI: 0.03, P = 0.004) were significantly positive compared with condition count. However, no significant differences for IDI and NRI were observed between MI and MIDC. Among Chinese older adults, weighted multimorbidity index with individual disease can better predict five-year mortality risk over condition count. There was little improvement in the predictive performance of the index after considering the joint effects of disease combinations.

